# Co-expression network of heat-response transcripts: A glimpse into how splicing factors impact rice basal thermotolerance

**DOI:** 10.3389/fmolb.2023.1122201

**Published:** 2023-02-02

**Authors:** Hadrien Georges Boulanger, Wenbin Guo, Lucca de Filipe Rebocho Monteiro, Cristiane Paula Gomes Calixto

**Affiliations:** ^1^ Université Paris-Saclay, Gif-sur-Yvette, France; ^2^ École Nationale Supérieure d'Informatique pour l'Industrie et l’Entreprise, Evry-Courcouronnes, France; ^3^ Department of Botany, Institute of Biosciences, University of São Paulo, São Paulo, Brazil; ^4^ Information and Computational Sciences, The James Hutton Institute, Dundee, United Kingdom

**Keywords:** *Oryza sativa* L., heat stress, alternative splicing, co-expression network analysis (WGCNA), eigengene, hierarchical clustering, systems biology

## Abstract

To identify novel solutions to improve rice yield under rising temperatures, molecular components of thermotolerance must be better understood. Alternative splicing (AS) is a major post-transcriptional mechanism impacting plant tolerance against stresses, including heat stress (HS). AS is largely regulated by splicing factors (SFs) and recent studies have shown their involvement in temperature response. However, little is known about the splicing networks between SFs and AS transcripts in the HS response. To expand this knowledge, we constructed a co-expression network based on a publicly available RNA-seq dataset that explored rice basal thermotolerance over a time-course. Our analyses suggest that the HS-dependent control of the abundance of specific transcripts coding for SFs might explain the widespread, coordinated, complex, and delicate AS regulation of critical genes during a plant’s inherent response to extreme temperatures. AS changes in these critical genes might affect many aspects of plant biology, from organellar functions to cell death, providing relevant regulatory candidates for future functional studies of basal thermotolerance.

## Introduction

Predicted increases in air temperatures threaten global food security (Teixeira et al., 2013; Redden et al., 2014). Therefore, it is important to understand the responses that allow plants to tolerate heat (Janni et al., 2020). In response to heat stress (HS), plants undergo massive changes in the transcriptome, proteome, sugar levels, membrane composition and rate of photosynthesis (Penfield, 2008; Zou et al., 2011; Su et al., 2018; Vitoriano and Calixto, 2021). Regarding the transcriptome, alternative splicing (AS) is a major gene-regulatory mechanism enhancing transcriptome and proteome diversity. As a result, AS allows for increased flexibility during responses to changing environmental conditions (Syed et al., 2012; Staiger and Brown, 2013; Filichkin et al., 2015). Several studies have contributed to our knowledge of crucial AS regulations upon HS (Jiang et al., 2017; Ling et al., 2018; Sanyal et al., 2018; Vitoriano and Calixto, 2021; Roces et al., 2022). For example, we analysed rice response to HS and identified 2,162 differentially alternatively spliced (DAS) genes, many of which code for key regulators of gene expression, confirming that AS is a major part of the HS response (Vitoriano and Calixto, 2021).

Common AS regulators are RNA binding proteins (RBPs), such as Ser/Arg-rich (SR) proteins, which then interact with the spliceosome (Kornblihtt et al., 2013). The level and activity of hundreds of these splicing regulators, also known as splicing factors (SFs), change in response to temperature, suggesting they are crucial elements in thermal-stress AS regulation (Verhage et al., 2017; Calixto et al., 2018; Vitoriano and Calixto, 2021). Our knowledge of the true scale and function of SFs involved in heat-induced AS are limited and need to be addressed (Rosenkranz et al., 2022). Genome-wide transcriptome data can offer vast amounts of valuable information, and a systems-oriented, network-based analysis of this data could be used to help decipher the molecular mechanisms behind stress responses (Raza, 2020; Winck et al., 2021). Co-expression networks, for example, are powerful systems biology tools that use expression datasets to predict candidate regulators, their targets and other important elements from biological systems (Coneva et al., 2014; Aghamirzaie et al., 2016; Zhang et al., 2022). Here, we generated a co-expression regulatory network from a publicly available RNA-seq dataset of the rice basal or inherent thermotolerance response (Luo et al., 2019) to explore potential heat-related AS regulators and their targets in a genome-wide context. This allowed us to have a mapping of the most influential SFs under HS, as well as their putative targets, which include genes involved in diverse functions, such as chloroplast development and cell death.

## Method

### Differential AS analysis with RNA-seq data

In our work, we used the SRP190858 RNA-seq dataset (Luo et al., 2019), which we have described previously (Vitoriano and Calixto, 2021). Briefly, this dataset contained data from the stem and leaves of Nipponbare rice plants grown for 2 weeks at 28°C and subjected to 45°C HS over 2 days. Three biological replicates were harvested at eight time points during the HS, namely at 0 h (28°C), 1 h after going 45°C, 3 h, 6 h, 12 h, 24 h, 36 h and 48 h. RNA-seq raw files were decompressed through fastq-dump (Bioconda) and trimmed with Trimmomatic (v0.36) (Bolger et al., 2014) in paired-read mode (Supplementary Figure S1). Salmon in paired-read mode (Patro et al., 2017) coupled with the rice Nipponbare reference transcriptome (Kawahara et al., 2013) was used to quantify rice transcripts in Transcript Per Million (TPM) (Supplementary File S1). In our previous study, we used the 3D RNA-seq App (Guo et al., 2021; Vitoriano and Calixto, 2021) to compare the gene and transcript expression between 0 h (28°C) and 24 h (45°C) for a heat-specific response, thus eliminating the diel variation as a factor. We identified 3,140 differential transcript usage (DTU) transcripts (Supplementary File S2). DTU transcripts are those that show significantly different expression changes when compared to the changes of the other transcripts of the same gene, which can be caused by AS regulation and not necessarily by transcriptional regulation. The genes with DTU transcripts were defined as the differentially alternatively spliced (DAS) genes (Vitoriano and Calixto, 2021).

### Normalisation, transcripts clustering and splicing network construction

Z-scores were calculated using expression data of DTU transcripts in TPM and used for clustering analysis. For this, we subtracted each transcript’s mean expression over all the time points to each expression data at a given time and divided the result by the standard deviation of this transcript’s expression over all the time points. We used WGCNA (v1.70.3) (Langfelder and Horvath, 2008) and the Z-scores of DTU transcripts to classify them into co-expressed clusters in response to HS. The transcript expression similarities for *hclust* clustering were optimised with the *power* parameter set as 17 following the elbow heuristic method on a scale-free property measure for our clustering 0.7 (Khanin and Wit, 2006). The obtained dendrogram was cut with the *dynamicTreeCut* package (Langfelder et al., 2008), with the *deepSplit* parameter set as 4–in order to have the most homogeneous clusters as well as the least transcripts in the outlier cluster (cluster 0), which was removed in further analysis.

For each SF/RBP-coding transcript of interest, correspondent TPM values were extracted and Z-scores were calculated. The identification of protein-coding transcripts was carried out using TranSuite, with default parameters (v0.2.3) (Entizne et al., 2020). We then calculated the Pearson correlations between cluster means (centroid) and SFs/RBPs normalised expression. This allowed us to create a bipartite graph visualised as a co-expression network in Cytoscape (Shannon et al., 2003). A minimum correlation threshold of 0.924293 (in absolute value) was chosen.

### *In silico* functional analysis

We explored the following databases: Phytozome for gene-related nucleotide sequences (Goodstein et al., 2012), RGAP (Kawahara et al., 2013) and Oryzabase (Kurata and Yamazaki, 2006) for gene functional reports, PLAZA for precomputed phylogenetic trees (Van Bel et al., 2022) and InterPro for the biological function of protein domains (Blum et al., 2021). Schematic diagrams of gene structures were made with the help of the Exon–Intron Graphic Maker program [http://wormweb.org/exonintron (accessed on 4th October 2022)]. Gene Ontology statistical overrepresentation test was carried out with Panther version 16.0 [http://pantherdb.org/(accessed on 26th October 2022)] with the binomial test and cut-off *p*-value < 0.5. Subcellular localization prediction was carried out with LOCALIZER 1.0.4 (Sperschneider et al., 2017) and WoLF PSORT (Horton et al., 2007).

## Results

### Construction of a HS response splicing-related network

To construct a rice HS splicing network, we used the RNA-seq time-course data published by Luo et al. (2019) (SRA dataset SRP190858). This dataset was chosen because it generated the highest number of DAS genes upon HS (1,633) when compared to other available datasets (the second highest had 678 genes) (Vitoriano and Calixto, 2021). Additionally, it has one of the highest sampling regimes of rice plants undergoing HS (0 h, 1 h, 3 h, 6 h, 12 h, 24 h, 36 h, and 48 h), which will increase the quality of our network. The experimental design of this RNA-seq dataset consisted of rice plants grown at 28°C for 2 weeks and later exposed to 45°C for 48 h. We previously investigated heat-responsive genes and transcripts in this dataset and found 3,140 DTU transcripts (Supplementary File S2) from 1,633 rice genes (Vitoriano and Calixto, 2021). To use the expression data of these DTU transcripts in the construction of our network, we grouped these transcripts into 19 different clusters using the WGCNA method (Supplementary File S2, Supplementary Figure S2).

**FIGURE 1:**
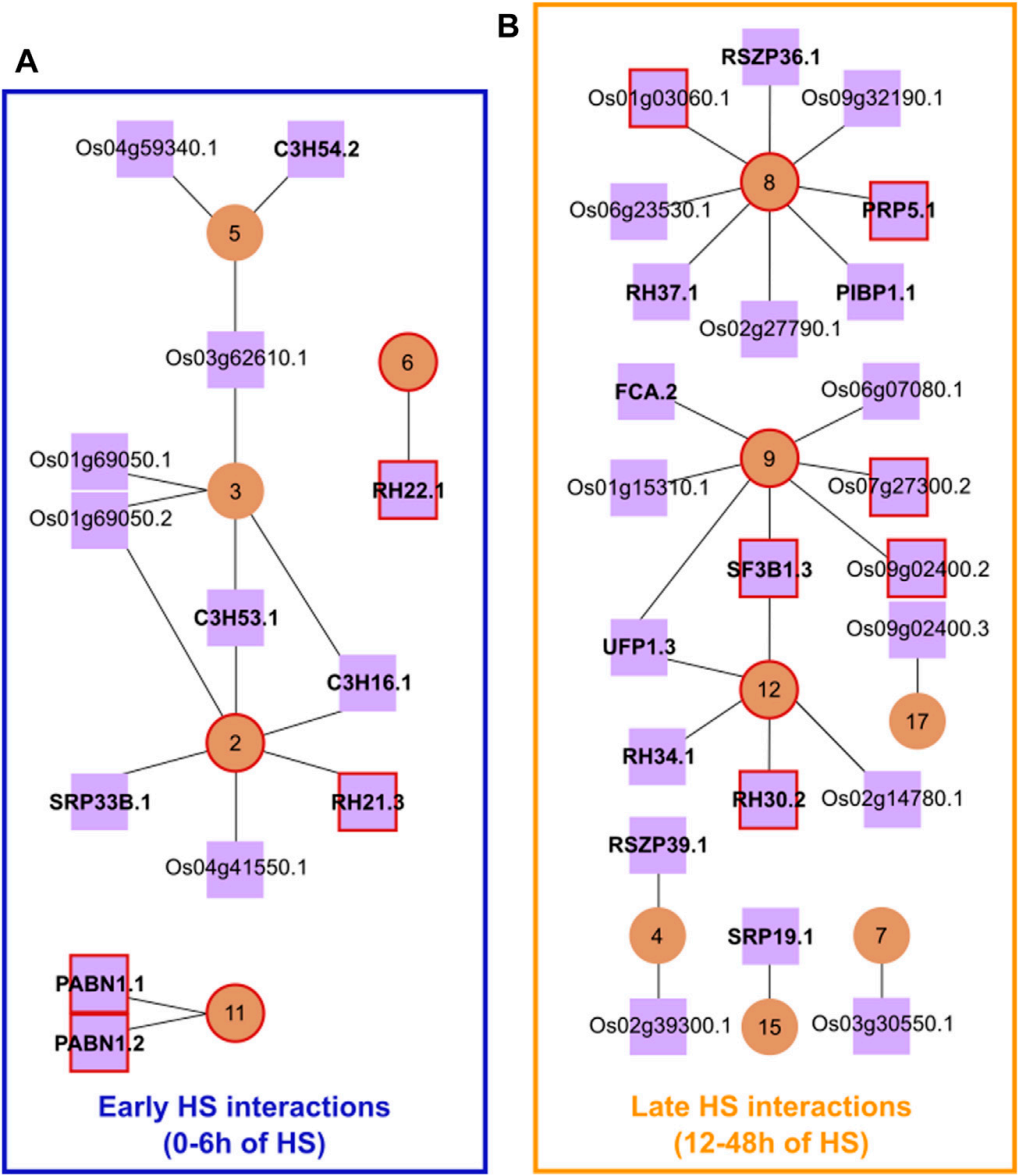
Rice HS response splicing network. Main interactions between DTU transcripts clusters (circular brown nodes) and SF/RBPs (squared lilac nodes). **(A)** Subnetworks framed in blue are the Early HS Interactions (0 h-6 h of HS), while **(B)** those framed in yellow are the Late HS Interactions (12 h-48 h of HS). Red-coloured circles/squares highlight the presence of DTU transcripts of the splicing regulators themselves in the marked clusters. Lilac nodes that are closely on top of each other represent transcript isoforms from the same gene. For clarity, the prefix LOC_ has been omitted from gene IDs in the figure and in this legend. Gene symbols in bold were taken from The Committee on Gene Symbolization, Nomenclature and Linkage (CGSNL). *C3H16.1, C3H53.1*, and *C3H54.2*: ZINC FINGER CCCH DOMAIN-CONTAINING PROTEIN 16/53/54 (Os02g35150.1, Os07g48410.1, and Os08g03310.2, respectively); *SRP33B.1*: SERINE/ARGININE-RICH PROTEIN 33B (Os07g47630.1); *RH21.3*, *RH22.1*, *RH30.2*, *RH34.1*, and *RH37.1*: RNA HELICASE 21/22/30/34/37 (Os03g50090.3, Os09g21520.1, Os01g68320.2, Os03g36930.1 and Os03g59050.1, respectively); *PABN1*: POLY **(A)** BINDING PROTEIN 1 (Os02g52140.1 and Os02g52140.2); *RSZP36.1* and *RSZP39.1*: RS DOMAIN WITH ZINC KNUCKLE PROTEIN 36/39 (Os05g02880.1 and Os05g07000.1, respectively); *PRP5.1*: RNA HELICASE PRP5 (Os08g06344.1); *PIBP1.1*: PIGMR-INTERACTING AND BLAST RESISTANCE PROTEIN 1 (Os03g50560.1); *FCA.2:* OSFCA PROTEIN (Os09g03610.2); *SF3B1.3*: CORE SPLICING FACTOR SF3B1 (Os02g05310.3); UFP1.3: UP-FRAMESHIFT1 (Os07g31340.3); *SRP19.1*, SIGNAL RECOGNITION PARTICLE 19 (Os06g23430.1). Network table containing original data used to draw the figure is found in Supplementary Table S1.

To identify the potential regulatory elements in our network, we selected a list of putative protein-coding transcripts of SFs and RBPs that were significantly differentially expressed (DE), DAS or both DE + DAS in the heat (Vitoriano and Calixto, 2021). From this list, we only kept transcripts coding for a functional protein with a nuclear localisation signal. As a result, we obtained 186 protein-coding SF/RBP transcripts, from 106 genes, with 48 SF/RBP genes having more than one protein-coding transcript (Supplementary File S3). Next, we correlated the cluster means of DTU transcript Z-scores with those of the Z-scores of individual protein-coding SF/RBP transcripts. To increase the robustness of our network, interactions–between clusters and putative splicing regulators–were filtered to include only those in the top 1% of correlation coefficients (Figure 1). This resulted in a network having only positive correlations. The network retained clusters 2-9, 11, 12, 15 and 17 as well as 36 protein-coding SF/RBP transcripts. A temporal pattern of expression was also observed among the clusters (Supplementary Figure S2): early changes, with up/down-regulation occurring within the first 6 h of HS, and late changes, with up-regulation occurring after ≥ 12 h of HS. To carry out further analysis, we divided the different subnetworks into two groups: Early HS Interactions and Late HS Interactions.

### Early HS interactions

The Early HS Interactions group, hereafter referred to as EI, has 17 strong correlations between DTU clusters and SF/RBPs, involving 1,212 transcripts from 1,011 genes. Some SF/RBPs have strong interactions with more than one cluster and *vice versa*. Yet, the HS response profiles were diverse, revealing a finer post-transcriptional control of genes over time (Supplementary Figure S2). For example, the SF/RBPs *C3H54.2*, Os04g59340.1 and Os03g62610.1 have strong associations with cluster 5 (253 DTU transcripts)–most of them show a fast downregulation of expression after 1 h of HS, which is stably kept at low levels on subsequent time points. This is an indication that one of these SF/RBPs could be involved in the DAS regulation of genes in this cluster. The SF/RBP RH22 could be involved with AS regulation of transcripts in cluster 6 (216 DTU transcripts)–most of them show a transient up-regulation of transcripts at around 6 h of HS, which is reduced again from 12 h of HS and is kept low until 48 h of HS. It is noteworthy that several genes have transcripts in different clusters. One reason for this is that these genes are targets of more than one splicing factor, undergoing a time-dependent AS regulation. These results open the possibility that the fast HS-dependent control of the abundance of specific protein-coding SF/RBP transcripts explains the rapid, widespread, coordinated, complex, and fine AS regulation in the early hours of exposure to extreme temperatures.

**FIGURE 2:**
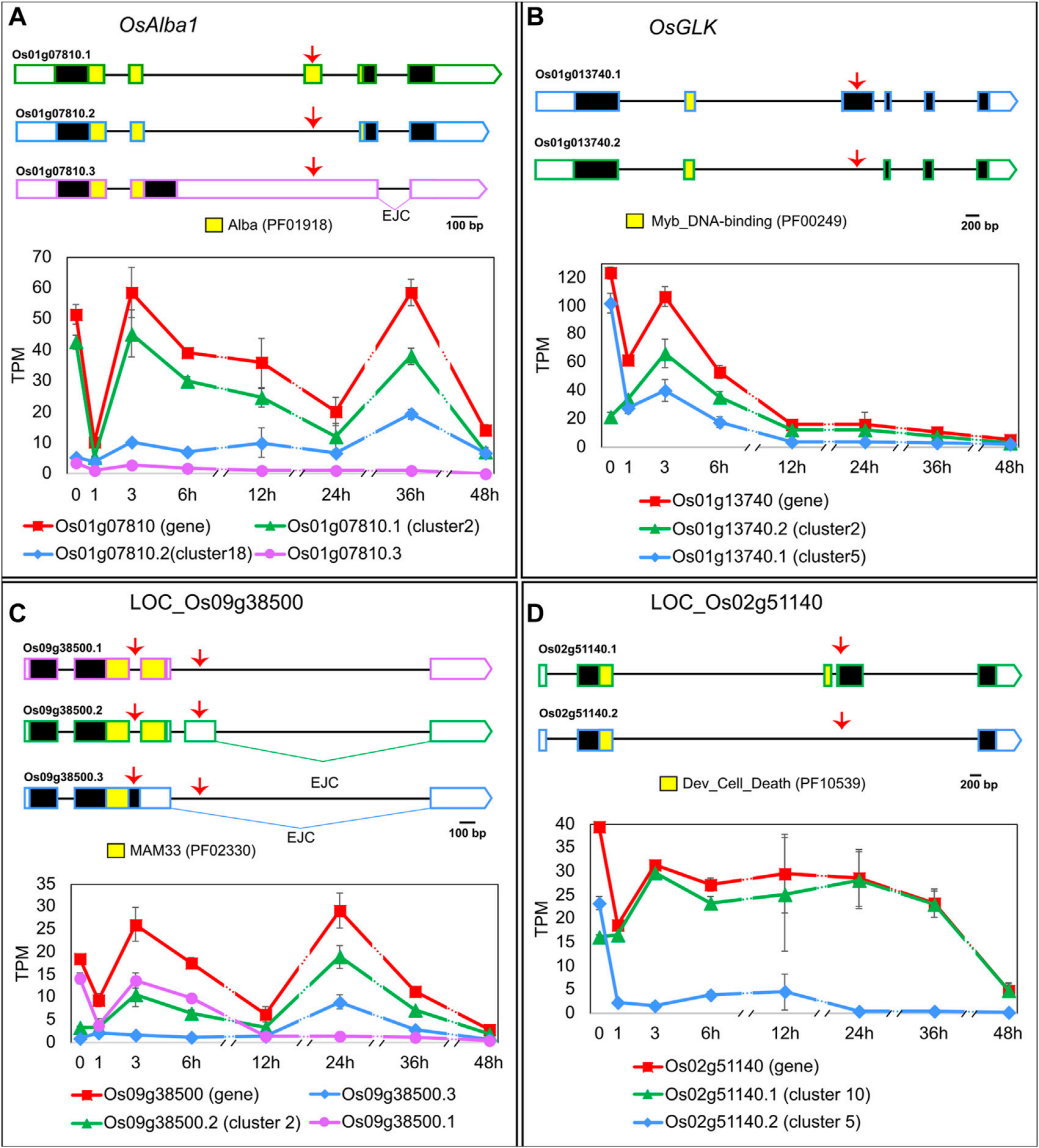
Heat-induced AS of **(A)** *OsAlba1* (LOC_Os01g07810), **(B)** *OsGLK* (LOC_Os01g13740), **(C)** LOC_Os09g38500 and **(D)** LOC_Os02g51140. 5′ and 3′ UTRs are open boxes; introns are represented with thin lines; coding sequences are shown as dark boxes, except for domain-encoding exons, which are coloured (Pfam accession ID in brackets). EJC: exon junction complex >50 nt downstream a stop codon. Alternative splicing events are marked with red arrows. X-axis: hours in HS (Luo et al., 2019). Error bars: standard error of the mean. For clarity, the prefix LOC_ was omitted from most gene IDs in the figure. Transcript LOC_Os01g07810.3 **(A)** was not mentioned in the text because it is expressed at very low levels throughout the experiment, compared to other transcripts of the same gene. Total gene expression level in TPM, shown in red, is a sum of all transcript abundances for each gene.

To learn which biological processes are being regulated by the EI, we carried out the following *in silico* analyses. A GO analysis with cluster 2 identified a significantly higher than expected number of genes related to “gene expression” (*p*-value < 0.001) and “mRNA processing” (*p*-value < 0.001), suggesting a role in transcriptional and post-transcriptional regulations. Indeed, EI’s clusters contain DTU transcripts from genes coding for SFs (e.g., LOC_Os05g48960: Splicing factor U2AF small subunit B), epigenetic-related proteins (e.g., LOC_Os09g35920: Mediator complex protein OsMED10) and TFs (example below), among others. The regulated heat-induced AS events either increased or decreased the relative levels of non-functional transcripts of protein-coding genes or altered the abundance of different protein-coding isoforms. For example, the OsACETYLATION LOWERS BINDING AFFINITY 1 (*OsAlba1*, LOC_Os01g07810) gene codes for a dehydration-responsive nuclear protein involved in stress tolerance (Verma et al., 2014; Verma et al., 2018). *OsAlba1* generates two protein isoforms–LOC_Os01g07810.1 in cluster 2 and LOC_Os01g07810.2 in cluster 18–that differ in the Alba domain and behave differently in response to HS (Figure 2A). Given that the Alba domain is related to nucleotide binding and target specificity (Aravind et al., 2003), the HS-induced alternative splicing of *OsAlba1* likely results in differential regulation of its targets. Another example is OsGOLDEN2-LIKE (*OsGLK*, LOC_Os01g13740.2), a transcription factor and homologue of *AtGLK1* and *AtGLK2*, both involved in chloroplast development. *OsGLK* generates two transcript isoforms coding for different proteins (one in cluster 2 and the other in cluster 5) that undergo an isoform switch at around 1 h of HS (Figure 2B). If these two OsGLK protein isoforms carry out different functions, the heat-induced isoform switch in *OsGLK* is likely to impact chloroplast development. Given that chloroplasts are involved in HS responses (Song et al., 2021), we further explored additional connections between EI clusters and this organelle. We found that cluster 3 has the highest proportion–83%–of transcripts coding for proteins with predicted chloroplast transit peptides and/or associated with plastid-related GO Cellular Component terms when compared to other clusters, suggesting a link between early AS regulation and chloroplast function. We also observed important AS events in mitochondria-related genes. For example, LOC_Os09g38500.1 (cluster 2) codes for a mitochondrial glycoprotein, probably responsible for protecting the mitochondrial membrane system from HS damage (Hu et al., 2022), while isoforms LOC_Os09g38500.2 and LOC_Os09g38500.3 have exon junction complexes > 50 nt downstream their stop codon, a frequent NMD-targeting feature (Hug et al., 2016), meaning they might not be translated. We observed that these three transcripts switch in their relative proportions upon different exposure times to high temperatures (Figure 2C), suggesting that LOC_Os09g38500 splicing regulation can be crucial for HS responses. As the last example, we observed a heat-induced isoform switch in LOC_Os02g51140 (Figure 2D), a gene coding for a development and cell death (DCD) domain-containing protein. In non-HS conditions, plants express isoforms LOC_Os02g51140.1, which codes for the fully functional DCD domain, and LOC_Os02g51140.2, which codes for a truncated DCD domain, in proportions around 41% and 59%, respectively, of the total gene expression. In 1 h of HS, isoform LOC_Os02g51140.1 becomes the most prevalent isoform, reaching > 99% at 48 h of HS, suggesting that HS-dependent AS regulation of LOC_Os02g51140 might be involved in HS-induced programmed cell death. In summary, early-induced AS changes in response to HS might affect many aspects of plant biology, from organellar functions to cell death.

### Late HS interactions

The Late HS Interactions (LI) has 25 strong correlations between DTU clusters and SF/RBPs, involving 1,090 transcripts from 901 genes. All clusters and SF/RBPs in LI show an upregulation of their transcripts at different times of the late HS response (Supplementary Figure S2). For example, transcripts *OsUPF1.3* (Os07g31340.3) and LOC_Os02g14780.1 have strong associations with cluster 12 (105 DTU transcripts)–most of them show stable expression levels in the first 12 h of HS, being upregulated only from 24 h of HS. Such co-expression indicates that proteins coded from these SF/RBP transcripts could regulate the DTU of cluster 12. LOC_Os09g02400.3 and cluster 17 (73 DTU transcripts) show a strong upregulation mostly at 48 h of HS. Several LI genes have DTU transcripts in different clusters, even EI clusters. One possible reason for this is that these genes are targets of more than one splicing factor. These analyses suggest that a delayed HS response is controlled by late expression changes of specific protein-coding SF/RBP transcripts.

**FIGURE 3:**
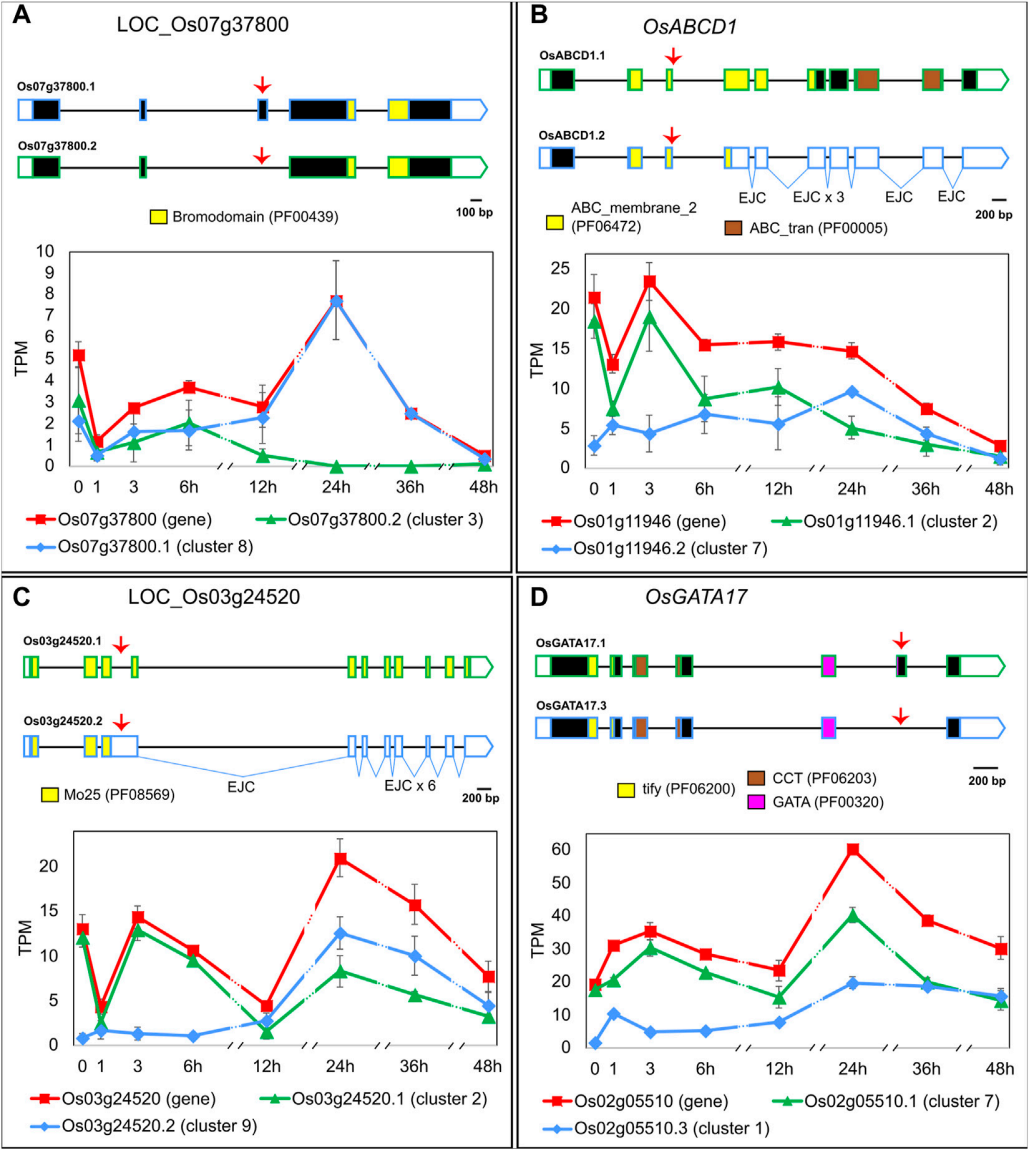
Heat-induced AS of **(A)** LOC_Os07g37800, **(B)** *OsABCD1* (LOC_Os01g11946), **(C)** LOC_Os03g24520 and **(D)** OsGATA17 (LOC_Os02g05510). 5′ and 3′ UTRs are open boxes; introns are represented with thin lines; coding sequences are shown as dark boxes, except for domain-encoding exons, which are coloured (Pfam accession ID in brackets). EJC: exon junction complex >50 nt downstream a stop codon. Alternative splicing events are marked with red arrows. X-axis: hours in HS (Luo et al., 2019). Error bars: standard error of the mean. For clarity, the prefix LOC_ was omitted from most gene IDs. Total gene expression level in TPM, shown in red, is a sum of all transcript abundances for each gene.

To explore the functional importance of regulating the late HS response through AS, we carried out the following *in silico* analyses with LI’s genes. Similarly to what we observed in EI, the AS events regulated in LI affect transcript stability and/or increase the protein-coding capacity of genes with diverse molecular functions. For example, LOC_Os07g37800, a gene that codes for a bromodomain-containing protein and whose Arabidopsis homologue (At1g61215) is involved in histone acetylation (Pandey et al., 2002), generates two transcript isoforms coding for different proteins, which might impact protein function (Figure 3A). In non-HS conditions and within the first 6 h of HS, both isoforms are expressed in similar proportions. As the exposure to heat is prolonged, isoform LOC_Os07g37800.1 becomes prevalent, reaching 100% at 24 h of HS. Another example is the ABC TRANSPORTER D FAMILY MEMBER 1 (*OsABCD1*, LOC_Os01g11946) gene, which generates two transcripts. Isoform *OsABCD1.1* codes for a protein involved in the peroxisomal import of fatty acids (Verrier et al., 2008), and it undergoes an isoform switch with PTC-containing isoform *OsABCD1.2* at 12 h–24 h of HS (Figure 3B). This suggests that late heat-induced AS of *OsABCD1* affects the relative amount of its functional transcripts, which likely impacts protein levels and, as a consequence, fatty acid transport. A similar AS regulation was observed for LOC_Os03g24520, a gene that codes for a Mo25-like domain-containing protein–a domain known to be involved in cell division (Mendoza et al., 2005). The proportion of the only protein-coding transcript, LOC_Os03g24520.1, is reduced from 93% of the total in non-HS conditions down to 37% at 12 h of HS (Figure 3C). As a result, LOC_Os03g24520 protein levels and, consequently, the rate of cell division, might be reduced by the slow heat-induced AS of this gene. Lastly, GATA TRANSCRIPTION FACTOR 17 (*OsGATA17*, LOC_Os02g05510), a TF that undergoes AS in response to abiotic stresses (Ye et al., 2009; Gupta et al., 2017), generates two transcript isoforms coding for proteins that differ towards the C-terminal region of the GATA domain, suggesting they could have different target genes. In non-HS conditions, rice Nipponbare plants mostly expressed isoform *OsGATA17.1* (90% of the total) but upon longer exposure to HS we observed a change in transcript proportions and both isoforms became equally expressed, which could impact gene function (Figure 3D). In summary, late AS changes upon prolonged exposure to extreme temperatures might affect many levels of the regulation of gene expression, from the epigenetic to metabolic levels, which consequently could have a profound impact on the cellular response to HS.

## Discussion

We have constructed a co-expression network based on transcriptomic data of rice leaves undergoing HS over 2 days. This network involves two kinds of nodes: protein-coding transcripts expressed from heat-sensitive genes that are also putative AS regulators (SF/RBPs) and clusters of transcripts regulated by AS (DTU transcript modules). An edge represents a strong correlation between these two kinds of nodes and we explored this correlation in terms of AS regulation. The advantages of our approach over a simpler gene-specific one are 1) AS regulation affects the proportion of transcripts expressed from a gene, so our DTU approach allows the analysis at the transcript level while gene-level analysis could not characterise AS regulations; 2) expression data of individual transcripts coding for SF/RBPs proteins are more likely to be associated with protein functionality, vis-à-vis splicing regulation, than gene expression levels, especially for genes with more than one transcript; 3) we are able to assume regulatory directions, i.e., from AS regulators to the regulated clusters–positive or negative regulation depending on the correlation coefficient; 4) working with clusters instead of individual DTU transcripts is less computationally intensive, while still allowing the construction of an effective and biologically meaningful network (Langfelder and Horvath, 2007).

Having mentioned the main advantages, we must also mention the major caveats of our approach. Firstly, correlation does not mean causation, so not all interactions will reflect AS regulation. For example, *OsFCA.2* (present in LI) is an unlikely AS regulator because if we take into consideration the expression of all transcripts coding for the same functional FCA protein, we no longer observe a strong correlation with cluster 9. In this case, the presence of an FCA transcript in cluster 10 suggests it is regulated by AS, rather than an AS regulator. Indeed, FCA post-transcriptional regulation is key in flowering time control (Du et al., 2006) and thermotolerance mechanisms (Lee et al., 2015). Secondly, not all DTU transcripts analysed here are exclusively regulated by AS mechanisms, e.g., they can also be regulated by epigenetic mechanisms, and RNA degradation, among others (Pajoro et al., 2017; Su et al., 2018). Lastly, SFs and other AS regulators act on specific cis-regulatory sequences of pre-mRNAs, dictating the final splicing outcome at the event level (Tognacca et al., 2020; Ganie and Reddy, 2021; Yang et al., 2022). Given that our network is rather transcript-specific, AS event regulation was not comprehensively covered in our work. Therefore, transcriptome regulatory mechanisms could be further explored in rice HS networks by additional studies on AS events and other gene regulatory mechanisms.

We identified potential AS regulators of several alternatively spliced rice genes in the early and late stages of the HS response, most of which are known SFs. For example, DEAD-box ATP-dependent RNA helicases are present in EI and LI. They belong to the largest family of enzymes with functions in RNA metabolism, including AS under stress conditions (Bourgeois et al., 2016; Lu et al., 2020; Terrone et al., 2022). The SF/RBPs *OsC3H16.1*, *OsC3H53.1*, and *OsSRP33B.1* could be involved in the heat-induced DAS regulation of genes in cluster 2. Indeed, members of the C3H gene family are part of the spliceosomal complex and likely play important roles in RNA processing control upon stress tolerance (Wang et al., 2008). OsSRP33B and its homologues in Arabidopsis (AtSR30) and grape (VvSR30) were shown to be specific splicing modulators upon environmental changes, regulating even their own splicing (Lopato et al., 1999; Isshiki et al., 2006; Jiang et al., 2017). *OsSRP33B* autoregulation, however, was not present in our 1% highest correlations splicing network (Pearson cut-off of ∼0.92), but we observed a 0.85 Pearson correlation between *OsSRP33B.1* and cluster 1, where transcript *OsSRP33B.8* is present, suggesting the autoregulation is possible. In the late response, the *OsUPF1* gene could be involved in AS regulation of genes in cluster 12. Its Arabidopsis orthologue, *AtUPF1* (At5g47010), encodes a protein required for non-sense-mediated mRNA decay and it was recently suggested that it could also have a role in splicing (Raxwal et al., 2020). The SR proteins encoded by transcripts *OsRSZP36.1* and *OsRSZP39.1* are strongly correlated with clusters 8 and 4, respectively. In this case, SR proteins are well-known SFs (Isshiki et al., 2006) strengthening our hypothesis that they regulate DTU transcripts of these clusters. Similarly, *SF3B1.3*, highly correlated with clusters 9 and 12, regulates intron retention and is involved in stress responses (Butt et al., 2021). The splicing network also contains RBPs whose functions are unknown or have not yet been associated with splicing, which is the case for *PIBP1,* LOC_Os03g30550 and LOC_Os02g39300, for example. The strong correlation between such RBPs and DTU clusters suggests their novel role in AS regulation, which merits further investigation. In summary, different splicing regulators have a role in the early and late rice HS response. This temporal regulation reinforces the well-known function of SFs in fine-tuning gene expression in response to environmental changes through AS (Jiang et al., 2017).

Our approach considerably facilitated the discovery of previously unknown candidates involved in the HS response and expanded our knowledge of known heat-responsive genes. For example, Hu et al. (2022) carried out a genome-wide association analysis that identified LOC_Os09g38500 as a candidate gene for thermotolerance. This gene is present in our network and we suggest that LOC_Os09g38500 splicing regulation (Figure 2C) can be crucial for HS responses. We also identified remarkable AS regulation increasing the protein-coding capacity of key photosynthesis-related genes, such as *OsGLK* (Figure 2B), suggesting AS involvement in the regulation of photosynthesis and chloroplast development in response to HS. This hypothesis is particularly interesting in light of recent studies on chloroplast retrograde signals (CRS), which is a plastid-to-nucleus signalling mechanism that can regulate nuclear gene expression, especially in response to stress, including HS (Song et al., 2021). CRS was shown to regulate AS of splicing factor *AtSR30* (Petrillo et al., 2014) and transcription of *ZmGLK1* (Kendrick et al., 2022). The rice orthologues of both *SR30* and *GLK* are present in our robust splicing network, as well as many other chloroplast-related genes, which, if taken together with the information on CRS, allow us to suggest the existence of an important AS- and CRS-dependent feedback loop of HS responses between the chloroplast and the nucleus. Another important mechanism involved in stress tolerance is programmed cell death (Tenhaken et al., 2005; Chua et al., 2019), and rice cultivar Nipponbare, analysed here, has a low basal thermotolerance, where plant death was observed in HS assays (Lin et al., 2014). In support of this, we observed an AS-dependent increased level of LOC_Os02g51140’s transcripts coding for the fully functional DCD domain-containing protein (Figure 2D), suggesting its involvement in the HS-induced programmed cell death. In conclusion, we propose that the putative HS-related role of many genes depicted in our network is dependent on AS regulation, and any further molecular and functional analysis of these genes should consider AS regulation.

Our examination of HS responses at the level of transcripts and its modules highlighted features that have been overlooked by previous studies. We incorporated novel information into an HS network that places post-transcriptional regulation, especially AS, into adaptive, physiological and developmental contexts, while also revealing a higher-order organisation of the transcriptome. This work has shown the imperativeness of AS analysis in genetic and molecular studies involving thermotolerance.

## Data Availability

The datasets presented in this study can be found in online repositories. The names of the repository/repositories and accession number(s) can be found in the article/Supplementary Material.

## Supplementary material

Supplementary file s1 Rice transcript expression in TPM.

Supplementary file s2 3140 DTU transcripts table.supplementary-material.

Supplementary file s3 Correlation between clusters and SF/RBP transcripts table.supplementary-material.

## References

[B1] Aghamirzaie D. Collakova E. Li S. Grene R. (2016). CoSpliceNet: CoSpliceNet: A framework for co-splicing network inference from transcriptomics data. BMC Genomics 17, 845. 10.1186/s12864-016-3172-6 27793091 PMC5086072

[B2] Aravind L. Iyer L. M. Anantharaman V. (2003). The two faces of Alba: The evolutionary connection between proteins participating in chromatin structure and RNA metabolism. Genome Biol. 4, R64. 10.1186/gb-2003-4-10-r64 14519199 PMC328453

[B3] Blum M. Chang H.-Y. Chuguransky S. Grego T. Kandasaamy S. Mitchell A. (2021). The InterPro protein families and domains database: 20 years on. Nucleic Acids Res. 49, D344–D354. 10.1093/nar/gkaa977 33156333 PMC7778928

[B4] Bolger A. M. Lohse M. Usadel B. (2014). Trimmomatic: Trimmomatic: A flexible trimmer for illumina sequence data. Bioinformatics 30, 2114–2120. 10.1093/bioinformatics/btu170 24695404 PMC4103590

[B5] Bourgeois C. F. Mortreux F. Auboeuf D. (2016). The multiple functions of RNA helicases as drivers and regulators of gene expression. Nat. Rev. Mol. Cell Biol. 17, 426–438. 10.1038/nrm.2016.50 27251421

[B6] Butt H. Bazin J. Alshareef S. Eid A. Benhamed M. Reddy A. S. N. (2021). Overlapping roles of spliceosomal components SF3B1 and PHF5A in rice splicing regulation. Commun. Biol. 4, 529. 10.1038/s42003-021-02051-y 33953336 PMC8100303

[B7] Calixto C. P. G. Guo W. James A. B. Tzioutziou N. A. Entizne J. C. Panter P. E. (2018). Rapid and dynamic alternative splicing impacts the Arabidopsis cold response transcriptome. Plant Cell 30, 1424–1444. 10.1105/tpc.18.00177 29764987 PMC6096597

[B8] Chua A. Fitzhenry L. Daly C. T. (2019). Sorting the wheat from the chaff: Programmed cell death as a marker of stress tolerance in agriculturally important cereals. Front. Plant Sci. 10. 1539, 10.3389/fpls.2019.01539 31850031 PMC6888703

[B9] Coneva V. Simopoulos C. Casaretto J. A. El-kereamy A. Guevara D. R. Cohn J. (2014). Metabolic and co-expression network-based analyses associated with nitrate response in rice. BMC Genomics 15, 1056. 10.1186/1471-2164-15-1056 25471115 PMC4301927

[B10] Du X. Du X. Qian X. Wang D. Du X. Qian X. (2006). Alternative splicing and expression analysis of OsFCA (FCA in Oryza sativa L.), a gene homologous to FCA in Arabidopsis. DNA Seq. 17, 31–40. 10.1080/10425170500136707 16753815

[B11] Entizne J. C. Guo W. Calixto C. P. G. Spensley M. Tzioutziou N. Zhang R. (2020). TranSuite: A software suite for accurate translation and characterization of transcripts. 10.1101/2020.12.15.422989

[B12] Filichkin S. Priest H. D. Megraw M. Mockler T. C. (2015). Alternative splicing in plants: Alternative splicing in plants: Directing traffic at the crossroads of adaptation and environmental stress. Curr. Opin. Plant Biol. 24, 125–135. 10.1016/j.pbi.2015.02.008 25835141

[B13] Ganie S. A. Reddy A. S. N. (2021). Stress-induced changes in alternative splicing landscape in rice: Functional significance of splice isoforms in stress tolerance. Biology 10, 309. 10.3390/biology10040309 33917813 PMC8068108

[B14] Goodstein D. M. Shu S. Howson R. Neupane R. Hayes R. D. Fazo J. (2012). Phytozome: A comparative platform for green plant genomics. Nucleic Acids Res. 40, D1178–D1186. 10.1093/nar/gkr944 22110026 PMC3245001

[B15] Guo W. Tzioutziou N. A. Stephen G. Milne I. Calixto C. P. Waugh R. (2021). 3D RNA-seq: 3D RNA-seq: A powerful and flexible tool for rapid and accurate differential expression and alternative splicing analysis of RNA-seq data for biologists. RNA Biol. 18, 1574–1587. 10.1080/15476286.2020.18582536286.2020.1858253 33345702 PMC8594885

[B16] Gupta P. Nutan K. K. Singla-Pareek S. L. Pareek A. (2022). Abiotic stresses cause differential regulation of alternative splice forms of GATA transcription factor in rice. Front. Plant Sci. 8. Available at: https://www.frontiersin.org/articles/10.3389/fpls.2017.01944 . 10.3389/fpls.2017.01944 PMC5693882 29181013

[B17] Horton P. Park K.-J. Obayashi T. Fujita N. Harada H. Adams-Collier C. J. (2007). WoLF PSORT: Protein localization predictorWoLF PSORT: Protein localization predictor. Nucleic Acids Res.Nucleic Acids Res. 35, W585–W587. 10.1093/nar/gkm259 17517783 PMC1933216

[B18] Hu C. Jiang J. Li Y. Song S. Zou Y. Jing C. 2022. QTL mapping and identification of candidate genes using a genome-wide association study for heat tolerance at anthesis in rice (*Oryza sativa* L). Front. Genet. 13, 983525, 10.3389/fgene.2022.983525 36186421 PMC9520461

[B19] Hug N. Longman D. Cáceres J. F. (2016). Mechanism and regulation of the nonsense-mediated decay pathway. Nucleic Acids Res. 44, 1483–1495. 10.1093/nar/gkw010 26773057 PMC4770240

[B20] Isshiki M. Tsumoto A. Shimamoto K. (2006). The serine/arginine-rich protein family in rice plays important roles in constitutive and alternative splicing of pre-mRNA. Plant Cell 18, 146–158. 10.1105/tpc.105.037069 16339852 PMC1323490

[B21] Janni M. Gullì M. Maestri E. Marmiroli M. Valliyodan B. Nguyen H. T. (2020). Molecular and genetic bases of heat stress responses in crop plants and breeding for increased resilience and productivity. J. Exp. Bot. 71, 3780–3802. 10.1093/jxb/eraa034 31970395 PMC7316970

[B22] Jiang J. Liu X. Liu C. Liu G. Li S. Wang L. (2017). Integrating omics and alternative splicing reveals insights into grape response to high temperature. Plant Physiol. 173, 1502–1518. 10.1104/pp.16.01305 28049741 PMC5291026

[B23] Kawahara Y. de la Bastide M. Hamilton J. P. Kanamori H. McCombie W. R. Ouyang S. (2013). Improvement of the Oryza sativa Nipponbare reference genome using next generation sequence and optical map data. Rice 6, 4. 10.1186/1939-8433-6-4 24280374 PMC5395016

[B24] Kendrick R. Chotewutmontri P. Belcher S. Barkan A. (2022). Correlated retrograde and developmental regulons implicate multiple retrograde signals as coordinators of chloroplast development in maize. Plant Cell 34, 4897–4919. 10.1093/plcell/koac276 36073948 PMC9709983

[B25] Khanin R. Wit E. (2006). How scale-free are biological networks. J. Comput. Biol. 13, 810–818. 10.1089/cmb.2006.13.810 16706727

[B26] Kornblihtt A. R. Schor I. E. Alló M. Dujardin G. Petrillo E. Muñoz M. J. (2013). Alternative splicing: Alternative splicing: A pivotal step between eukaryotic transcription and translation. Nat. Rev. Mol. Cell Biol. 14, 153–165. 10.1038/nrm3525 23385723

[B27] Kurata N. Yamazaki Y. (2006). Oryzabase. An integrated biological and genome information database for rice. Plant Physiol. 140, 12–17. 10.1104/pp.105.063008 16403737 PMC1326027

[B28] Langfelder P. Horvath S. (2007). Eigengene networks for studying the relationships between co-expression modules. BMC Syst. Biol. 1, 54. 10.1186/1752-0509-1-54 18031580 PMC2267703

[B29] Langfelder P. Horvath S. (2008). Wgcna: Wgcna: an R package for weighted correlation network analysis. BMC Bioinforma. 9, 559. 10.1186/1471-2105-9-559 PMC2631488 19114008

[B30] Langfelder P. Zhang B. Horvath S. (2008). Defining clusters from a hierarchical cluster tree: The dynamic tree cut package for R. Bioinforma. Oxf. Engl. 24, 719–720. 10.1093/bioinformatics/btm563 18024473

[B31] Lee S. Lee H.-J. Jung J.-H. Park C.-M. (2015). The *Arabidopsis thaliana* RNA-binding protein FCA regulates thermotolerance by modulating the detoxification of reactive oxygen species. New Phytol. 205, 555–569. 10.1111/nph.13079 25266977

[B32] Lin M. Chai K. Ko S. Kuang L. Lur H.-S. Charng Y. (2014). A positive feedback loop between HEAT SHOCK PROTEIN101 and HEAT STRESS-ASSOCIATED 32-KD PROTEIN modulates long-term acquired thermotolerance illustrating diverse heat stress responses in rice varieties. Plant Physiol. 164, 2045–2053. 10.1104/pp.113.229609 24520156 PMC3982761

[B33] Ling Y. Serrano N. Gao G. Atia M. Mokhtar M. Woo Y. H. (2018). Thermopriming triggers splicing memory in Arabidopsis. J. Exp. Bot. 69, 2659–2675. 10.1093/jxb/ery062 29474581 PMC5920379

[B34] Lopato S. Kalyna M. Dorner S. Kobayashi R. Krainer A. R. Barta A. (1999). atSRp30, one of two SF2/ASF-like proteins from *Arabidopsis thaliana*, regulates splicing of specific plant genes. Genes Dev. 13, 987–1001. 10.1101/gad.13.8.987 10215626 PMC316644

[B35] Lu C.-A. Huang C.-K. Huang W.-S. Huang T.-S. Liu H.-Y. Chen Y.-F. (2020). DEAD-box RNA helicase 42 plays a critical role in pre-mRNA splicing under cold stress. Plant Physiol. 182, 255–271. 10.1104/pp.19.00832 31753844 PMC6945872

[B36] Luo Y. Fang B. Wang W. Yang Y. Rao L. Zhang C. (2019). Genome-wide analysis of the rice J-protein family: Identification, genomic organization, and expression profiles under multiple stresses. 3 Biotech. 9, 358. 10.1007/s13205-019-1880-8 PMC6730974 31544012

[B37] Mendoza M. Redemann S. Brunner D. (2005). The fission yeast MO25 protein functions in polar growth and cell separation. Cell Biol. 84, 915–926. 10.1016/j.ejcb.2005.09.013 16325501

[B38] Pajoro A. Severing E. Angenent G. C. Immink R. G. H. (2017). Histone H3 lysine 36 methylation affects temperature-induced alternative splicing and flowering in plants. Genome Biol. 18, 102. 10.1186/s13059-017-1235-x 28566089 PMC5452352

[B39] Pandey R. Müller A. Napoli C. A. Selinger D. A. Pikaard C. S. Richards E. J. (2002). Analysis of histone acetyltransferase and histone deacetylase families of *Arabidopsis thaliana* suggests functional diversification of chromatin modification among multicellular eukaryotes. Nucleic Acids Res. 30, 5036–5055. 10.1093/nar/gkf660 12466527 PMC137973

[B40] Patro R. Duggal G. Love M. I. Irizarry R. A. Kingsford C. (2017). Salmon provides fast and bias-aware quantification of transcript expression. Nat. Methods 14, 417–419. 10.1038/nmeth.4197 28263959 PMC5600148

[B41] Penfield S. (2008). Temperature perception and signal transduction in plants. New Phytol. 179, 615–628. 10.1111/j.1469-8137.2008.02478.x 18466219

[B42] Petrillo E. Herz M. A. G. Fuchs A. Reifer D. Fuller J. Yanovsky M. J. (2014). A chloroplast retrograde signal regulates nuclear alternative splicing. Science 344, 427–430. 10.1126/science.1250322 24763593 PMC4382720

[B43] Raxwal V. K. Simpson C. G. Gloggnitzer J. Entinze J. C. Guo W. Zhang R. (2020). Nonsense-mediated RNA decay factor UPF1 is critical for posttranscriptional and translational gene regulation in Arabidopsis. Plant Cell 32, 2725–2741. 10.1105/tpc.20.00244 32665305 PMC7474300

[B44] Raza A. (2020). Metabolomics: A systems biology approach for enhancing heat stress tolerance in plants. Plant Cell Rep. 41, 741–763. 10.1007/s00299-020-02635-8 33251564

[B45] Redden R. J. Hatfield J. L. Vara Prasad P. V. Ebert A. W. Yadav S. S. O’Leary G. J. (2014). “Temperature, climate change, and global food security,” in temperature and plant development (john wiley & sons, ltd), 181–202. 10.1002/9781118308240.ch8

[B46] Roces V. Lamelas L. Valledor L. Carbó M. Cañal M. J. Meijón M. (2022). Integrative analysis in Pinus revealed long-term heat stress splicing memory. Cell Mol. Biol. 112, 998–1013. 10.1111/tpj.15990 PMC9828640 36151923

[B47] Rosenkranz R. R. E. Ullrich S. Löchli K. Simm S. Fragkostefanakis S. (20222022). Relevance and regulation of alternative splicing in plant heat stress response: Current understanding and future directions. Front. Plant Sci. 13, 911277. 10.3389/fpls.2022.911277 PMC9260394 35812973

[B48] Sanyal R. P. Misra H. S. Saini A. (2018). Heat-stress priming and alternative splicing-linked memory. J. Exp. Bot. 69, 2431–2434. 10.1093/jxb/ery111 29718462 PMC5920290

[B49] Shannon P. Markiel A. Ozier O. Baliga N. S. Wang J. T. Ramage D. (2003). Cytoscape: Cytoscape: A software environment for integrated models of biomolecular interaction networks. Genome Res. 13, 2498–2504. 10.1101/gr.1239303 14597658 PMC403769

[B50] Song Y. Feng L. Alyafei M. A. M. Jaleel A. Ren M. (2021). Function of chloroplasts in plant stress responses. Int. J. Mol. Sci. 22, 13464. 10.3390/ijms222413464 34948261 PMC8705820

[B51] Sperschneider J. Catanzariti A.-M. DeBoer K. Petre B. Gardiner D. M. Singh K. B. (2017). Localizer: Localizer: Subcellular localization prediction of both plant and effector proteins in the plant cell. Sci. Rep. 7, 44598. 10.1038/srep44598 28300209 PMC5353544

[B52] Staiger D. Brown J. W. S. (2013). Alternative splicing at the intersection of biological timing, development, and stress responses. Plant Cell 25, 3640–3656. 10.1105/tpc.113.113803 24179132 PMC3877812

[B53] Su Z. Tang Y. Ritchey L. E. Tack D. C. Zhu M. Bevilacqua P. C. (2018). Genome-wide RNA structurome reprogramming by acute heat shock globally regulates mRNA abundance. Proc. Natl. Acad. Sci. 115, 12170–12175. 10.1073/pnas.1807988115 30413617 PMC6275526

[B54] Syed N. H. Kalyna M. Marquez Y. Barta A. Brown J. W. S. (2012). Alternative splicing in plants – coming of age. Trends Plant Sci. 17, 616–623. 10.1016/j.tplants.2012.06.001 22743067 PMC3466422

[B55] Teixeira E. I. Fischer G. van Velthuizen H. Walter C. Ewert F. (2013). Global hot-spots of heat stress on agricultural crops due to climate change. Agric. For. Meteorol 170, 206–215. 10.1016/j.agrformet.2011.09.002

[B56] Tenhaken R. Doerks T. Bork P. (2005). Dcd – A novel plant specific domain in proteins involved in development and programmed cell death. BMC Bioinforma. 6, 169. 10.1186/1471-2105-6-169 PMC1182354 16008837

[B57] Terrone S. Valat J. Fontrodona N. Giraud G. Claude J.-B. Combe E. 2022. RNA helicase-dependent gene looping impacts messenger RNA processing. Nucleic Acids Res., gkac717. 10.1093/nar/gkac717 PMC9458439 36039747

[B58] Tognacca R. S. Kubaczka M. G. Servi L. Rodríguez F. S. Herz G. Petrillo E. (2020). Light in the transcription landscape: Chromatin, RNA polymerase II and splicing throughout *Arabidopsis thaliana*’s life cycle. Transcription 11, 117–133. 10.1080/21541264.2020.17964731264.2020.1796473 32748694 PMC7714448

[B59] Van Bel M. Silvestri F. Weitz E. M. Kreft L. Botzki A. Coppens F. (2022). Plaza 5.0: Plaza 5.0: Extending the scope and power of comparative and functional genomics in plants. Nucleic Acids Res. 50, D1468–D1474. 10.1093/nar/gkab1024 34747486 PMC8728282

[B60] Verhage L. Severing E. I. Bucher J. Lammers M. Busscher-Lange J. Bonnema G. (2017). Splicing-related genes are alternatively spliced upon changes in ambient temperatures in plants. PLOS ONE 12, e0172950. 10.1371/journal.pone.0172950 28257507 PMC5336241

[B61] Verma J. K. Gayali S. Dass S. Kumar A. Parveen S. Chakraborty S. (2014). OsAlba1, a dehydration-responsive nuclear protein of rice (Oryza sativa L. ssp. indica), participates in stress adaptation. Phytochemistry 100, 16–25. 10.1016/j.phytochem.2014.01.015 24534105

[B62] Verma J. K. Wardhan V. Singh D. Chakraborty S. Chakraborty N. (2018). Genome-wide identification of the Alba gene family in plants and stress-responsive expression of the rice Alba genes. Genes 9, 183. 10.3390/genes9040183 29597290 PMC5924525

[B63] Verrier P. J. Bird D. Burla B. Dassa E. Forestier C. Geisler M. (2008). Plant ABC proteins – A unified nomenclature and updated inventory. Trends Plant Sci. 13, 151–159. 10.1016/j.tplants.2008.02.001 18299247

[B64] Vitoriano C. B. Calixto C. P. G. (2021). Reading between the lines: Reading between the lines: RNA-seq data mining reveals the alternative message of the rice leaf transcriptome in response to heat stress. Plants 10, 1647. 10.3390/plants10081647 34451692 PMC8400768

[B65] Wang D. Guo Y. Wu C. Yang G. Li Y. Zheng C. (2008). Genome-wide analysis of CCCH zinc finger family in Arabidopsis and rice. BMC Genomics 9, 44. 10.1186/1471-2164-9-44 18221561 PMC2267713

[B66] Winck F. V. Monteiro L. de F. R. Souza G. M. (2021). “Introduction: Advances in plant omics and systems biology,” in advances in plant omics and systems biology approaches”. Adv. Exp. Med. Biol. 1346, 1–9. 10.1007/978-3-030-80352-0_1 35113393

[B67] Yang X. Jia Z. Pu Q. Tian Y. Zhu F. Liu Y. (2022). ABA mediates plant development and abiotic stress via alternative splicing. Int. J. Mol. Sci. 23, 3796. 10.3390/ijms23073796 35409156 PMC8998868

[B68] Ye H. Du H. Tang N. Li X. Xiong L. (2009). Identification and expression profiling analysis of TIFY family genes involved in stress and phytohormone responses in rice. Plant Mol. Biol. 71, 291–305. 10.1007/s11103-009-9524-8 19618278

[B69] Zhang Y. Han E. Peng Y. Wang Y. Wang Y. Geng Z. (2022). Rice co-expression network analysis identifies gene modules associated with agronomic traits. Plant Physiol. 190, 1526–1542. 10.1093/plphys/kiac339 35866684 PMC9516743

[B70] Zou J. Liu C. Chen X. (2011). Proteomics of rice in response to heat stress and advances in genetic engineering for heat tolerance in rice. Plant Cell Rep. 30, 2155–2165. 10.1007/s00299-011-1122-y 21769604

