# Supplementary material for: Co-expression network of heat-response transcripts: A glimpse into how splicing factors impact rice basal thermotolerance
Source: Front Mol Biosci. 2023 Feb 2;10:1122201. doi: 10.3389/fmolb.2023.1122201 (PMC9932781; doi:10.3389/fmolb.2023.1122201)
Supplement: Supplementary file 2 [file Table1.PDF]

Supplementary Table 1. Human-readable rice HS response splicing network table.

SF/RBP transcript	Cluster	Correlation
**LOC_Os02g35150.1**	cluster_2	0.933414
**LOC_Os03g50090.3**	cluster_2	0.946368
**LOC_Os07g47630.1**	cluster_2	0.929623
**LOC_Os07g48410.1**	cluster_2	0.943028
**LOC_Os02g35150.1**	cluster_3	0.927412
**LOC_Os03g62610.1**	cluster_3	0.937433
**LOC_Os07g48410.1**	cluster_3	0.929616
**LOC_Os02g39300.1**	cluster_4	0.943247
**LOC_Os05g07000.1**	cluster_4	0.926486
**LOC_Os03g62610.1**	cluster_5	0.946539
**LOC_Os04g59340.1**	cluster_5	0.964084
**LOC_Os08g03310.2**	cluster_5	0.957158
**LOC_Os09g21520.1**	cluster_6	0.929346
**LOC_Os03g30550.1**	cluster_7	0.946519
**LOC_Os01g03060.1**	cluster_8	0.917215
**LOC_Os02g27790.1**	cluster_8	0.947858
**LOC_Os03g50560.1**	cluster_8	0.944466
**LOC_Os03g59050.1**	cluster_8	0.928076
**LOC_Os05g02880.1**	cluster_8	0.927234
**LOC_Os06g23530.1**	cluster_8	0.946515
**LOC_Os08g06344.1**	cluster_8	0.994984
**LOC_Os09g32190.1**	cluster_8	0.918423
**LOC_Os01g15310.1**	cluster_9	0.950915
**LOC_Os02g05310.3**	cluster_9	0.925126
**LOC_Os06g07080.1**	cluster_9	0.922724
**LOC_Os07g27300.2**	cluster_9	0.944436
**LOC_Os07g31340.3**	cluster_9	0.922798
**LOC_Os09g02400.2**	cluster_9	0.971851
**LOC_Os09g03610.2**	cluster_9	0.955034
**LOC_Os02g52140.1**	cluster_11	0.948277
**LOC_Os02g52140.2**	cluster_11	0.96211
**LOC_Os01g68320.2**	cluster_12	0.916634
**LOC_Os02g05310.3**	cluster_12	0.924293
**LOC_Os02g14780.1**	cluster_12	0.955171
**LOC_Os03g36930.1**	cluster_12	0.915899
**LOC_Os07g31340.3**	cluster_12	0.951562
**LOC_Os06g23430.1**	cluster_15	0.918501
**LOC_Os09g02400.3**	cluster_17	0.948117
