# Supplementary material for: Co-expression network of heat-response transcripts: A glimpse into how splicing factors impact rice basal thermotolerance
Source: Front Mol Biosci. 2023 Feb 2;10:1122201. doi: 10.3389/fmolb.2023.1122201 (PMC9932781; doi:10.3389/fmolb.2023.1122201)
Supplement: Supplementary file 3 [file Presentation1.pdf]

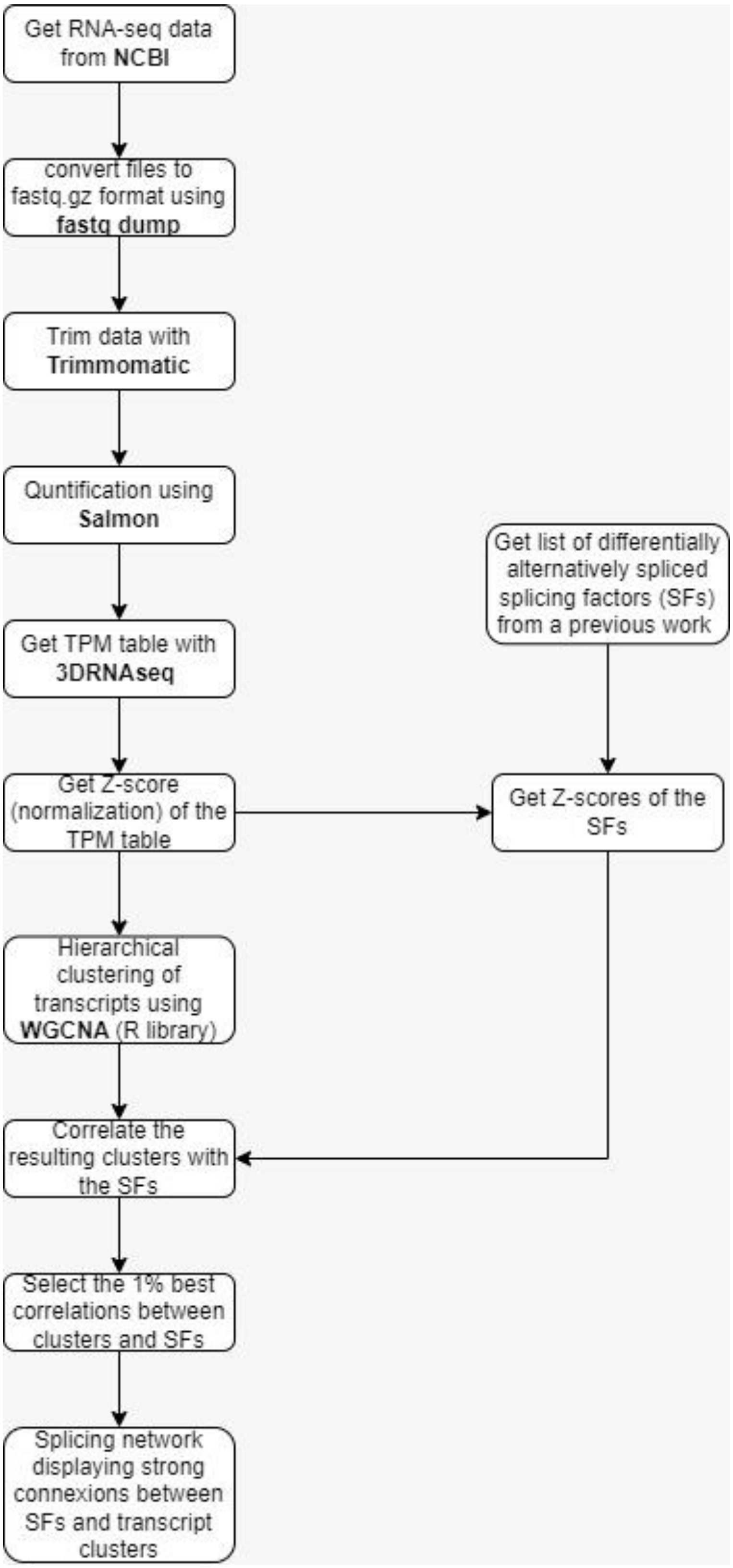

Supplementary Figure 1. Flow diagram of the main bioinformatics analysis carried out in our study.

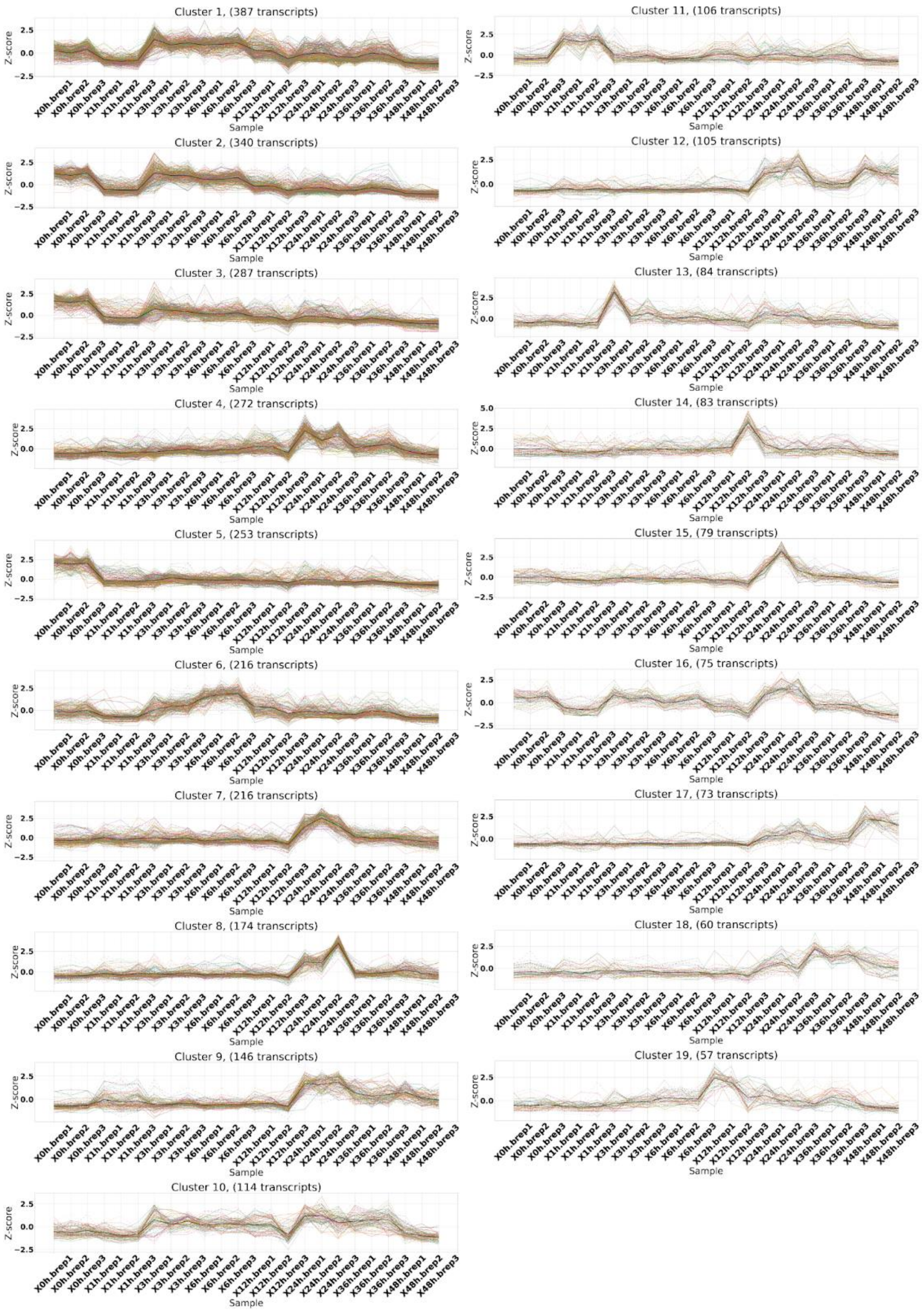

Supplementary Figure 2. Expression of DTU transcripts from 19 clusters. y-axis: expression z-score values; x-axis: 24 RNA-seq samples from 8 time-points and 3 biological replicates in each timepoint. The cluster number and the number of transcripts in the cluster are given.
